# Serpentine locomotion through elastic energy release

**DOI:** 10.1098/rsif.2017.0055

**Published:** 2017-05-31

**Authors:** F. Dal Corso, D. Misseroni, N. M. Pugno, A. B. Movchan, N. V. Movchan, D. Bigoni

**Affiliations:** 1 DICAM—University of Trento, via Mesiano 77, Trento, Italy; 2 Laboratory of Bio-Inspired and Graphene Nanomechanics, via Mesiano 77, Trento, Italy; 3 School of Engineering and Materials Science, Queen Mary University of London, London, UK; 4 Italian Space Agency, Via del Politecnico snc, Rome, Italy; 5 Department of Mathematical Sciences, University of Liverpool, Liverpool, UK

**Keywords:** elastica, configurational force, motility

## Abstract

A model for serpentine locomotion is derived from a novel perspective based on concepts from configurational mechanics. The motion is realized through the release of the elastic energy of a deformable rod, sliding inside a frictionless channel, which represents a snake moving against lateral restraints. A new formulation is presented, correcting previous results and including situations never analysed so far, as in the cases when the serpent's body lies only partially inside the restraining channel or when the body has a muscle relaxation localized in a small zone. Micromechanical considerations show that propulsion is the result of reactions tangential to the frictionless constraint and acting on the snake's body, a counter-intuitive feature in mechanics. It is also experimentally demonstrated that the propulsive force driving serpentine motion can be directly measured on a designed apparatus in which flexible bars sweep a frictionless channel. Experiments fully confirm the theoretical modelling, so that the presented results open the way to exploration of effects, such as variability in the bending stiffness or channel geometry or friction, on the propulsive force of snake models made up of elastic rods.

## 1. Introduction

Since the beginning of the research on creeping locomotion, four different mechanisms for snake motion have been identified [1–4]: serpentine, concertina, side-winding and rectilinear. A special feature characterizes the first of the four movements, namely that the snake has to exert *normal* forces against projections from the ground^1^ to induce longitudinal sliding, so that tangential frictional forces operating between the serpent and the terrain have to be minimized, because they simply *oppose* gliding forward. This model for locomotion, pioneered by Gray [1–3], is based on the muscular elasticity of the snake's body and allows us to explain how locomotion can occur in the *absence of tangential frictional forces*. The model is in agreement with the experimental observation that serpentine motion becomes impossible when a snake is confined to a straight or circular channel where variations in the elastic energy cannot occur. The mechanical model of Gray is made up of a chain of rigid pieces connected to each other with rotational springs (a set-up also employed for undulatory motion in a fluid with obstacles [8]), while a more refined model in which a snake is represented by an elastic rod (Euler's elastica; see, for instance, [9–11]) has been introduced in an almost unknown article by Kuznetsov *et al.* [12], based on results presented in another forgotten article by Lavrentiev and Lavrentiev [13]. The model of a deforming elastic rod has been independently considered later in other studies [5,14,15], including sand-swimming [16] and soft robotics [17].^2^

Recently, a way to achieve motion through a release of elastic energy has been related to the generation of configurational or ‘Eshelby-like’ forces [24–27] and its understanding has led to the discovery of torsional locomotion [28]. A novel reconsideration of the mechanics of snake motion as modelled by Kuznetsov *et al.* [12] has evidenced that their solution is inconsistent at some points (to be detailed later) and does not explain the generation of forces along the body of the snake. Furthermore, an experimental direct validation of the rod model has never been attempted. Therefore, a generalization and a rigorous derivation is presented in this article for the mechanics of an elastic rod sliding into a perfectly smooth channel, together with its experimental validation.

The propulsive force is independently derived using two approaches: (i) an energy formulation and (ii) an approach based on the ‘micromechanical’ derivation of all forces acting on the system, enhanced by the integration of the equations of motion. These two approaches have different merits, so that only the knowledge of both provides a complete picture of serpentine motion through a perfectly smooth channel. In particular, the energy formulation explains how configurational forces provide propulsion from a release of elastic energy. On the other hand, the micromechanical approach shows that *the perfectly frictionless channel provides propulsion by means of tangential localized reactions acting against the snake's body*. This is a surprising behaviour, counter-intuitive from a mechanical point of view.

Results are also presented for situations in which the snake is only partially inserted in the channel (a case never considered before), while the rest of the body does not have any restraints for propulsion. It is shown that a snake idealized as an elastic rod can always exit, but never enter, a frictionless channel. Finally, circumstances are also considered for the first time in which a discontinuity is present in the channel curvature or in the bending stiffness of the elastic rod simulating the snake. The latter case may be achieved by the snake with a localized muscular relaxation along the body, which is also interesting for snake robots or in the design of a colonoscope or other endoscopes [29]. To provide a direct validation of the theoretical model, a channel has been designed and realized, with the shape of a clothoid spiral, in which friction has been reduced to a negligible amount through the use of roller bearings. A series of experiments carried out in the ‘Instability Laboratory’ of the University of Trento, Italy, on elastic rods with uniform and variable bending stiffness are shown to fully confirm the theoretical results (see also the video in the electronic supplementary material). Although the presented experiments are limited to a channel in the form of a clothoid spiral and to three different types of elastic rods, the methodology introduced allows exploration of more complex situations, for instance involving friction at the rod/channel contact or sophisticated variability in the snake's bending stiffness or channel geometry.

## 2. The propulsion of a rod within a channel

### 2.1. Formulation of the problem

An elastic inextensible rod of length *l* is considered, rectilinear in its undeformed configuration, and with variable bending stiffness *B*(*s*), so that *s* is the curvilinear coordinate along the rod's axis, *s* ∈ [0, *l*], and represents a non-inertial reference frame. For generality, the bending stiffness will be assumed to be possibly discontinuous at discrete points.

**Figure 1.**
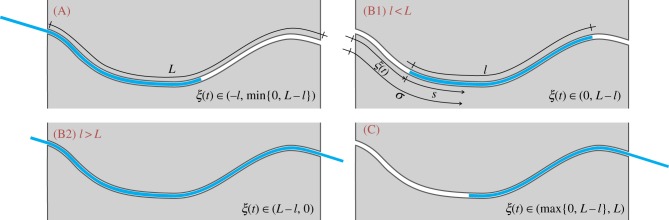
An inextensible elastic rod (rectilinear in its undeformed configuration) of length *l* is moving within a frictionless and smooth curvilinear channel. Its configuration is defined through the evolution in time of the curvilinear coordinate *ξ*(*t*), which singles out the position of the left end. Four cases arise: (A) and (C) where one of the rod's ends is inside the channel while the other remains unrestrained; (B1) where the whole rod is inside the channel and (B2) where the rod occupies the whole channel. (Online version in colour.)

The elastic rod (or a part of it) is moving inside a curved, frictionless (rigid and smooth) channel with curvilinear length *L*. The position of the rod's left end is singled out by the curvilinear coordinate *ξ*(*t*) ∈ [ − *l*, *L*], which is a function of the time *t* and measures the distance from the left end of the channel (figure 1).

In figure 1, the possible stages of the rod sliding within the channel are sketched at increasing values of the configurational parameter *ξ*(*t*), as defined in table 1:

— case (A), where the left part of the rod is unconstrained while the right end moves within the channel;
— cases (B1) and (B2), where the rod is entirely within the channel in the former case (possible only when *l* < *L*) and the rod has both ends external to the channel in the latter case (possible only when *l* > *L*);
— case (C), which is the opposite configuration of that described in case (A).

**Table 1.** Ranges of the configurational parameter *ξ* and values of the coordinates *s*_1_ and *s*_2_, together with their respective derivatives, for the four cases.

case	A	B1	B2	C
*ξ*(*t*)	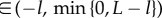	∈ (0, *L* − *l*)	∈ (*L* − *l*, 0)	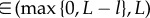
*s*_1_(*t*)	−*ξ*(*t*)	0	−*ξ*(*t*)	0
*s*_2_(*t*)	*l*	*l*	*L* − *ξ*(*t*)	*L* − *ξ*(*t*)
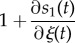	0	1	0	1
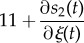	1	1	0	0

Denoting by a superimposed dot the time derivative, 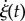 and 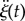 represent the velocity and the tangential acceleration of the rod's left end and also of all the points along the rod, because the rod is inextensible. The curvature *κ* of the rod axis depends on the global coordinate *σ*(*s*, *t*) = *ξ*(*t*) + *s* and corresponds to the curvature *χ* of the channel at each point *s* of the rod within the channel, while it is assumed null for the part of the elastic rod outside the channel, namely2.1
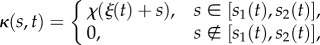
where *s*_1_ and *s*_2_ define, respectively, the initial and final curvilinear coordinate of the part of rod constrained by the channel,2.2
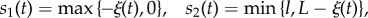
so that the four cases shown in figure 1 correspond to the pairs {*s*_1_(*t*), *s*_2_(*t*)} reported in table 1.

According to the Euler–Bernoulli model, the elastic rod reacts to bending with a moment *M* linearly proportional to the curvature *κ*, so that *M*(*s*, *ξ*(*t*)) = *B*(*s*)*κ*(*s* + *ξ*(*t*)). Any part of the elastic rod outside the channel is simply unloaded and therefore remains straight.

### 2.2. Energy approach: elastic energy turning into kinetic

At the generic time *t* of the motion of the inextensible elastic rod inside the channel, the system is characterized by the elastic flexural energy 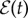 stored within the rod,2.3
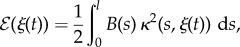
and the kinetic energy 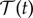 of the rod, which (assuming negligible rotational inertia) can be written as2.4
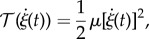
where 
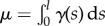
 is the total mass of the rod and *γ*(*s*) is its linear mass density.

Since the rigid channel is frictionless, the total potential energy of the system,^3^ 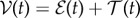, is constant in time, so that its time derivative vanishes2.5
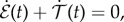
which leads to2.6
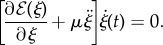
Defining the propulsive force *P*(*t*) as2.7
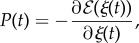
the equation of the longitudinal motion of the rod within the channel follows in the form2.8
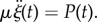
Note from equation (2.7) that the propulsive force *P* is a ‘resultant configurational force’, which is different from zero only when a change in the elastic strain energy 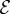 of the rod occurs through a change in the configurational parameter *ξ*, a situation similar to the Eshelby-like forces in elastic systems constrained by a sliding sleeve [26].

The two types of jump discontinuity are now introduced within the part of the rod constrained by the channel. One discontinuity is in the bending stiffness *B*(*s*) of the elastic rod at the local coordinate *s* = *l*_1_ ∈ (*s*_1_, *s*_2_)2.9
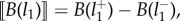
and the other discontinuity is in the curvature of the channel at the global coordinate *σ* = *L*_1_ ∈ (*ξ* + *s*_1_, *ξ* + *s*_2_)2.10
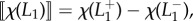
where the dependence on time has been omitted for simplicity (and also in the following). It is noted that the superscripts + and − define, respectively, the one-sided limit of the relevant quantity from the negative or positive direction.

Considering that the elastic energy (2.3) can be rewritten as2.11
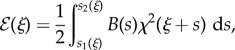
which is an integral with moving limits *s*_1_(*ξ*) and *s*_2_(*ξ*), and that the derivatives of the curvature field *χ*(*ξ* + *s*) with respect to *ξ* and *s* are identical,2.12
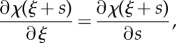
the propulsive force (2.7) can be calculated by means of the Leibniz integral rule as2.13
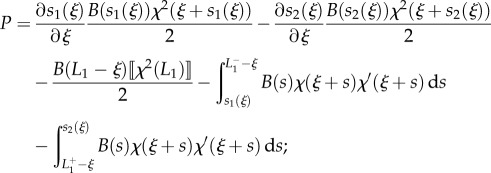
or, equivalently, after integration by parts, as2.14
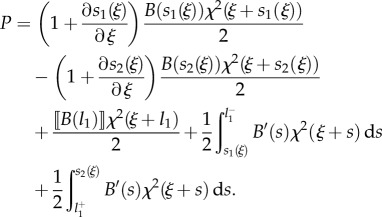
Equations (2.13) and (2.14) are fully equivalent and provide the same value of the propulsive force *P* for the given geometry and stiffness properties. However, the two formulae are expressed in a way that only one type of discontinuity appears. Indeed, the former (the latter) expression highlights the influence of only jump discontinuities in the squared channel curvature 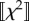 (in the bending stiffness 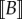), while the discontinuity in the stiffness (in the channel curvature) does not explicitly appear.

Specifying equation (2.14) for the four cases shown in figure 1, and considering the values of the derivatives ∂*s*_*j*_(*ξ*)/∂*ξ* (*j* = 1, 2) as computed in table 1, the propulsive force *P* simplifies to

— case A2.15
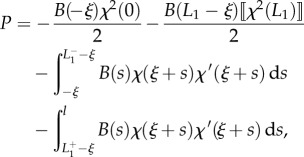
or, equivalently,2.16
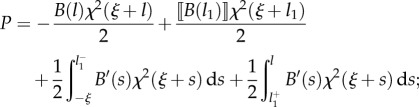
— case B12.17
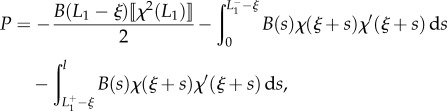
or, equivalently,2.18
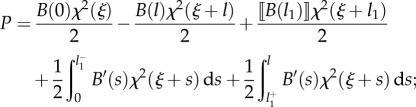
— case B22.19
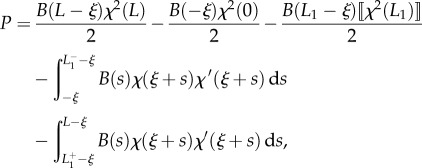
or, equivalently,2.20
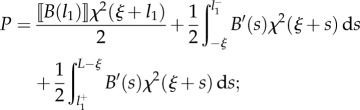
— case C2.21
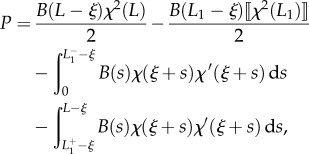
or, equivalently,2.22
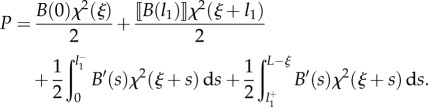

Equations (2.15)–(2.22) provide the theoretical evaluation of the propulsive force *P* that may be analytically or numerically computed for given distributions of bending stiffness *B*(*s*) along the rod and curvature of the channel *χ*(*σ*). It is worth mentioning that only the case B1 was previously analysed in [12] under several restrictive hypotheses (continuous channel curvature and rod bending stiffness, null bending moment and axial force at the end of the rod) so that equation (2.18) reduces to the result presented in [12] when 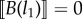. Note that equations (2.7) and (2.10) of [12] have incorrect signs. In addition, the normal forces internal to the snake have been erroneously disregarded in equation (2.7) of [30].

### 2.3. Propulsion from Newton's second law and micromechanics

Equations (2.13) and (2.14) provide the propulsive force in a global sense. This force is the result of the channel tangential reactions against the rod (which could be thought to be null at a first—mistaken—glance because the channel is frictionless), the knowledge of which is fundamental in the determination of the stress state in the snake's body during motion. These reactions, which have until now only been used to analyse the special case of the concentrated force at the end of the rod within a channel [5], can be derived with consideration of the dynamics of the rod's element and of the micromechanics of the elastic rod at its ends, at the entrance or the exit of the channel and at discontinuity points in the channel curvature or the rod's bending stiffness.

**Figure 2.**
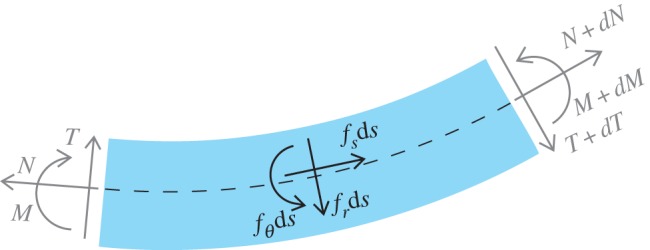
Free body diagram of an element of the elastic rod in the deformed configuration, where *M*(*s*), *T*(*s*) and *N*(*s*) denote bending moment, shear and axial forces, respectively, while *f*_*r*_(*s*), *f*_*s*_(*s*) and *f*_*θ*_(*s*) are the distributed external forces (as the sum of the real and apparent forces) applied to the rod, respectively, in the transverse, axial and rotational directions. (Online version in colour.)

The equilibrium equations for an elementary portion of a planar rod in a curved configuration (figure 2), defined by the curvature *κ*(*s*), can be written in the non-inertial frame of reference attached to the rod (at its left end, the origin of the curvilinear coordinate *s*) as2.23
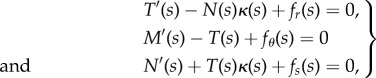
where *T*(*s*), *N*(*s*) and *M*(*s*) are the shear and axial forces, and the bending moment, respectively, while *f*_*r*_(*s*), *f*_*s*_(*s*) and *f*_*θ*_(*s*) are the distributed (total) forces applied to the rod, respectively, in the transverse, axial and rotational directions. By means of D'Alembert's principle, the equations of motion for an elementary portion of the rod can be obtained from equation (2.23) by considering the distributed forces *f*_*r*_(*s*), *f*_*s*_(*s*) and *f*_*θ*_(*s*) to be the sum of the real distributed forces (provided by the contact with the channel) and the apparent distributed forces (given by the negative of the inertia forces per unit length). More specifically

— for the part of the rod constrained by the channel, *κ*(*s*) = *χ*(*s*), and neglecting the rotational inertia of the rod, the distributed forces are given by2.24
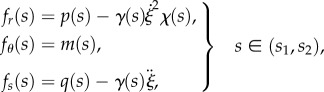
where *p*(*s*), *q*(*s*) and *m*(*s*) represent the channel reactions applied to the rod, respectively, in the transverse, axial and rotational directions;
— for the part of the rod outside the channel, this is assumed to remain straight (flexural vibrations are disregarded), *κ*(*s*) = 0, so that the distributed forces are given by2.25
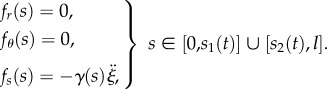
In this case, considering null boundary conditions at the ends, the integration of the equations of motion leads to 2.26
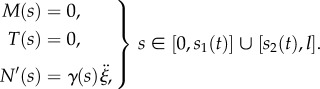

It is worth remarking that, in the case of quasi-static motion (namely, negligible inertial density forces), the distributed forces *f*_*r*_(*s*), *f*_*θ*_(*s*), *f*_*s*_(*s*) would all be either null or coincident with the channel reactions *p*(*s*), *m*(*s*), *q*(*s*), respectively, for the parts of the rod outside and within the channel.

#### 2.3.1. Micromechanics of a rod inside the channel

The tangential reactions acting at the rod's ends, at the channel entrance/exit, at the points of discontinuity of the channel curvature or of the rod's bending stiffness are derived here. These counter-intuitive reactions are strictly related to the deformability of the constrained rod and to the non-rectilinearity of the channel and have been found in other ‘non-classical’ situations [26,28,31].

**Figure 3.**
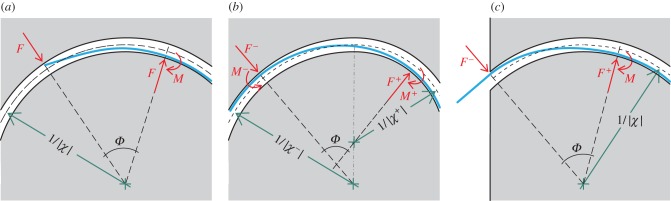
Micromechanics of an elastic rod inside a frictionless rigid channel with a small clearance. Forces developing at one end of the rod (*a*), at a point of jump in curvature from *χ*^−^ to *χ*^+^ (*b*) and at the edge of the channel (*c*). Note that the detached part of the rod has arc-length *a* and is singled out by the angle *Φ*. In the limit of null clearance, the detachment region vanishes and the two reactions orthogonal to the frictionless channel provide a resultant tangential force. (Online version in colour.)

Relaxing for a moment the assumption that the rod is perfectly tight to the channel, a clearance is introduced in the channel constraining the elastic rod as shown in figure 3. Owing to the presence of the gap, assumed small, between the elastic rod and the channel, the former always has a zone of detachment (singled out by the angle *Φ* and having arc-length *a*) from the latter. In the micromechanical analysis developed below it is shown that in the limit when the gap vanishes, so that the detachment region vanishes too, a non-null tangential reaction is developed by the frictionless channel.

In figure 3, three possible cases of interest are sketched, namely: (i) the rod's end is inside the channel; (ii) the channel curvature has a discontinuity; and (iii) the rod's end is outside the channel. In all cases, the reaction force *F* is orthogonal to the channel, which is frictionless. Below, these three cases will be analysed together with the further case in which (iv) the rod has a discontinuity in the bending stiffness.

#### Tangential reaction at the rod end inside the channel

The situation when one end of the elastic rod is inside the frictionless channel with radius of curvature 1/*χ* is sketched in figure 3*a*.

It is evident that the rod end is subject to the channel reaction *F* only, orthogonal to the upper contour of the channel. This force provides the moment *M* = *ΦF*/*χ* at the point (at the distance *a* = *Φ*/*χ* from the rod's end) where the elastic rod becomes adherent to the profile, so that *M* = *Bχ*. At that point the internal axial action can be approximated as *F* sin*Φ* ≈ *FΦ*, which becomes *B* *χ*^2^, the resultant axial force developing at the rod's end within a channel.

With reference to the curvilinear coordinate system *s*, if the left and the right ends of the rod are inside the channel, *s*_1_ = 0 and *s*_2_ = *l*, the compressive reaction forces are realized by the channel at the edges of the rod2.27
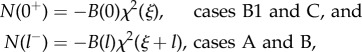
which have also been obtained using the principle of virtual power in [5] and are similar to the tangential reaction developing in a perfectly frictionless curvilinear profile constraining a clamped end of the rod [31].

#### Tangential reaction at a discontinuity point in the channel curvature

In the case when there is a jump in the curvature along the channel (figure 3*b*), the curvature of the rod takes a value different from the channel curvature because a small gap is present. In particular, the rod has a transition length along which its curvature passes from the channel curvature before to that after the jump. With reference to figure 3*b*, two concentrated contact reactions *F*^−^ and *F*^+^ develop orthogonal to the upper and lower contours of the channel at the rod's two detachment points. Owing to a difference in the angle *Φ* in the direction of the two reactions, a tangential resultant is expected. At equilibrium, in the limit of vanishing detachment length *a*, the two reactions orthogonal to the channel tend to the same value, *F*^+^ = *F*^−^ = *F*, and the tangential resultant is given by *F* sin*Φ* ≈ *FΦ*. The bending moments at the two detachment points are defined by the relevant curvature imposed by the channel, so that they are given by *M*^−^ = *Bχ*^−^ and *M*^+^ = *Bχ*^+^. With reference to the linear elastic solution of a clamped rod of length *a* and loaded at its free end by both a force *F* and a moment *M*^−^, the rotation angle at the loaded edge is *Φ* = *Fa*^2^/(2*B*) + *M*^−^ *a*/*B*. Since it follows by equilibrium that *Fa* = *M*^+^ − *M*^−^, the tangential resultant *FΦ* due to the jump in curvature 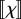 provides a concentrated tangential reaction equal to 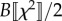. Therefore, with reference to a jump in curvature occurring at the point *s* = *L*_1_ − *ξ*, the presence of the above-mentioned tangential reaction provides a jump in the internal axial force corresponding to2.28
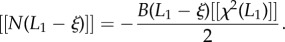


#### Tangential reaction at an exit point from the channel

The analysis of the previous case is also relevant to the condition when a rod's end is outside the channel (figure 3*c*). Indeed, in this case *χ*^−^ = 0 and, defining *χ*^+^= *χ*, the tangential resultant of the contact forces becomes *Bχ*^2^/2. In the special case of partial insertion of the rod into the channel, *s*_1_ ≠ 0 and *s*_2_ ≠ *l*, the tangential resultant *Bχ*^2^/2 provides the following jumps in the internal axial force:2.29
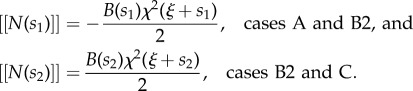
Note the following.

— The presence of the factor 1/2 distinguishes equation (2.29) from equation (2.27), which holds for the case of a rod entirely within the channel, *s*_1_ = 0 and *s*_2_ = *l*.
— The tangential reactions (2.29) have an expression similar to, but different in sign from, the Eshelby-like force generated at a sliding (straight) sleeve providing the reaction moment *M* = *Bχ* [26].

#### Tangential reaction at a point of discontinuity in the rod stiffness

At the point *s* = *l*_1_, where a jump in the bending stiffness 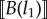 is present, a tangential concentrated contact reaction 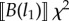 is developed in order to satisfy the rotational equilibrium of the infinitesimal part of the rod taking the centre of the osculating circle as the pivot point. This tangential reaction provides the following jump in the internal axial force:2.30
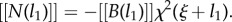


#### 2.3.2. Integration of the equations of motion

Considering the force distributions (2.24) and (2.25), the longitudinal inertia density 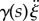 can be obtained from the equilibrium equations, equation (2.23), as2.31
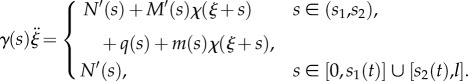


Considering now the possibility of discontinuities in the stiffness and in the curvature, respectively, at the coordinates *l*_1_ and *L*_1_ − *ξ* (equations (2.9) and (2.10)), integration of the longitudinal inertia density (equation (2.31)), over the length of the rod leads to the determination of the propulsive force *P* as2.32
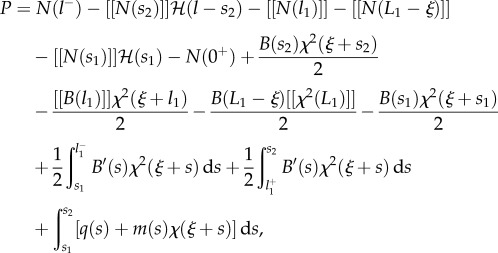
where2.33
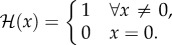
Although at first sight the propulsive force *P* given by equation (2.32) may appear different from that obtained through the energy approach (equation (2.14)), the two expressions are identical. Indeed, as shown through micromechanical concepts, (i) the jumps in the internal axial action 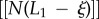 and 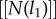 are given by equations (2.28) and (2.30), respectively, as well as (ii) the following identities (to be compared with equations (2.27) and (2.29)) hold:2.34
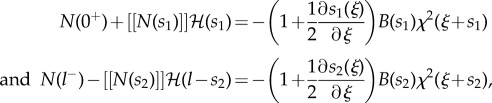
and, furthermore, due to the definition of a perfectly smooth channel,^4^ (iii) the virtual work done by the reaction forces *q*(*s*) and *m*(*s*) is null (at least in an integral sense),2.35
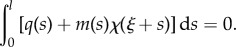


## 3. Examples

### 3.1. The case of a circular channel and the case of a rod with constant stiffness

The cases in which either the channel curvature or the rod stiffness is constant are considered.

In the case that a rod, with non-constant stiffness *B*(*s*), is constrained by a channel with constant curvature *χ*(*σ*) = *χ*, the propulsive force (2.14) reduces to3.1
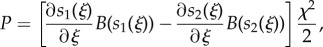
while in the case that a rod with constant stiffness *B*(*s*) = *B* is inside a channel, with non-constant curvature *χ*(*σ*), the propulsive force (2.14) reduces to3.2
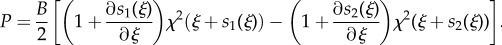


With reference to the possible four cases sketched in figure 1, the propulsive force simplifies as reported in table 2.

**Table 2.** Expression for the propulsive force *P* for the four possible cases (A, B1, B2 and C; figure 1) under the assumption of a channel with constant curvature *χ* (first row) or of a rod with constant bending stiffness *B* (second row).

case	A	B1	B2	C
*P* for const. curvature *χ*	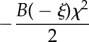	0	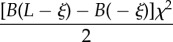	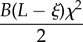
*P* for const. stiffness *B*	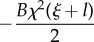	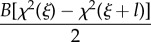	0	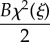

The evaluation of the propulsive force *P* in the different cases shows that:

— in the case when the rod has only one end outside the channel, an outward propulsive force is always realized in order to eject the rod from the channel on the side where the rod is initially out, namely 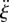 for case A and 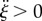 for case C;
— in the case of a channel with constant curvature, the propulsive force is null, *P* = 0, when the rod is completely inserted in the channel (case B1) so that the rod does not move; if both ends of the rod are outside the channel (case B2), an outward propulsive force is realized ejecting the rod away from the exit point of the channel where the rod has the highest bending stiffness;
— in the case of a rod with constant bending stiffness, the propulsive force is null, *P* = 0, when the rod has both ends outside the channel (case B2) so that the rod does not move; in the case when the rod is completely inserted in the channel, the propulsive force moves the rod away from the end deformed with the highest (in absolute value) curvature and towards the end deformed with the smallest one;
— in the case of a rod with constant bending stiffness constrained inside a channel with constant curvature the propulsive force is null, *P* = 0, whenever both the ends are constrained inside the channel (case B1) or both are free (case B2).

### 3.2. The two-piecewise constant rod stiffness inside a two-piecewise constant channel curvature

Let us now consider the case of both rod stiffness and channel curvature described by two-piecewise constant functions as follows:3.3
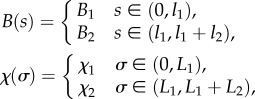
where *l*_1_ + *l*_2_ = *l*, *L*_1_ + *L*_2_ = *L* and for simplicity it is assumed that {*L*_1_, *L*_2_} > *l*.

**Figure 4.**
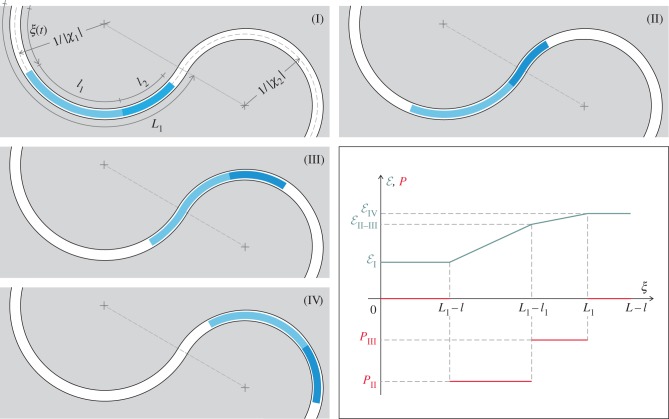
With reference to case B1 (figure 1), four subcases (I, II, III and IV) are depicted for a rod of piecewise constant bending stiffness constrained inside a channel of piecewise constant curvature, at increasing curvilinear coordinate *ξ*(*t*) defined as in table 3. The elastic energy 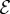 (green) and the propulsive force *P* (red) are plotted as functions of the curvilinear coordinate *ξ*. The plot is representative of the case |*χ*_1_| < |*χ*_2_| and *B*_1_ < *B*_2_. (Online version in colour.)

Restricting attention to the case of a rod completely inserted inside the channel (case B1 in figure 1), namely *ξ* ∈ (0, *L* − *l*), four possible subcases depending on the number of configurational parameters *ξ*(*t*) arise. The four subcases are sketched in figure 4 and listed in table 3 together with the elastic energy 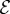 of the system and the propulsive force *P* evaluated in each case.

**Table 3.** Elastic energy 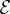 and propulsive force *P* for the four possible subcases (I, II, III and IV) of a two-piecewise constant bending stiffness rod completely inserted within a two-piecewise constant curvature channel (figure 4).

subcase	I	II	III	IV
*ξ*(*t*)	∈ (0, *L*_1_ − *l*)	∈ (*L*_1_ − *l*, *L*_1_ − *l*_1_)	∈ (*L*_1_ − *l*_1_, *L*_1_)	∈ (*L*_1_, *L* − *l*)
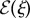	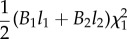	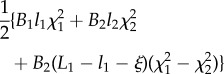	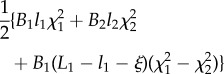	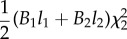
*P*	0	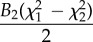	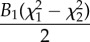	0

It can be concluded that

— the propulsive force is null, *P* = 0, in both subcases (I) and (IV) because a small perturbation in the configuration does not provide any change in the elastic energy of the system; this also occurs in subcases (II) and (III) in the special case when the two curvatures assume the same absolute value, |*χ*_1_| = |*χ*_2_|;
— a non-null propulsive force is generated in subcases (II) and (III) in the direction of a decreasing amount of rod constrained by the highest curvature (in absolute value).

With reference to the case |*χ*_1_| < |*χ*_2_| and *B*_1_ < *B*_2_, the propulsive force *P* and the strain energy 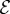 are reported in figure 4 as functions of the configurational parameter *ξ*. The value 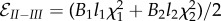 is introduced to define the strain energy at the transition from subcase II to subcase III. Note that subcases II and III provide a negative propulsive force, so that the motion is characterized by 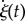, towards minimization of the elastic energy.

## 4. Experiments on snake propulsion with an elastic rod

**Figure 5.**
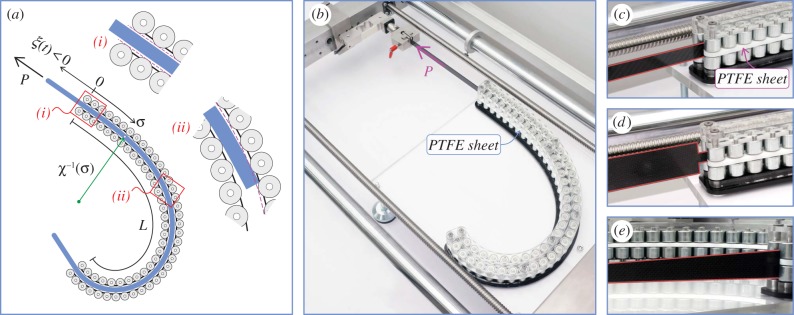
The design and realization of the frictionless channel, showing: (*a*) the Teflon layer interposed between rollers (note the detail of the end of the rod), (*b*) a photo of the apparatus during a test, and a series of details showing the Teflon (PTFE) sheet and the pairs of rollers constraining a rod having (*c*) constant stiffness, (*d*) a jump in stiffness and (*e*) a linearly varying stiffness. (Online version in colour.)

The experimental validation of the theoretical modelling presented in the previous sections was provided by employing the experimental set-up illustrated in figure 5. A frictionless channel, of length *L* = 750 mm, was designed and manufactured at the Instability Laboratory in the shape of a clothoid spiral. This spiral, used in road design to provide the best smooth transition between different tracks, was selected to be the ideal candidate for experiments because its curvature *χ* changes linearly with the arc length *σ*, so that *χ*(*σ*) = *π* *σ*/*a*^2^, which is the reason why the clothoid is considered the basic form assumed by a snake during its motion [30]. This curve is parametrically expressed through Fresnel integrals as [32]4.1
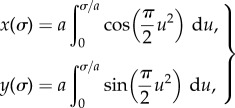
where *a* represents a scaling parameter. In the design of the smooth channel, a scaling parameter *a* = 510 mm was assumed, so that the curvature varies between 0 and 0.009 mm^−1^.

The two sides of the channel between which the elastic rod slides were realized with pairs of superimposed rollers (Misumi Europe; press-fit straight type, 20 mm diameter and 15 mm length), separated by a 3 mm thick Teflon (PTFE) sheet, which was introduced to reduce the discontinuities created by the gaps between the rollers (having rotation axis spaces of 21.62 mm). Two pairs of 66 and 76 rollers were used, respectively, for the short and long side of the channel, as shown in figure 5.

Different elastic rods were shaped by cutting (with an MDX-540 milling machine; Roland) ‘plain’ carbon fibre strips (*E* = 80148 MPa, *ρ* = 1620 kg m^−3^), 2.05 ± 0.05 mm thick, 40 mm wide and 2000 mm long. A tolerance of 0.5 mm was kept between the carbon rod and the rollers along the channel.

### 4.1. Ejection experiments

Qualitative experiments were performed by inserting (by hand) a rod of uniform bending stiffness into the channel and then suddenly releasing it. The release of elastic energy produces a violent upward ejection of the rod from the channel.

For a carbon-fibre rod of dimensions *b* = 16.2 mm, *t* = 1.35 mm, *l* = 660 mm, a mean exit velocity (calculated considering two subsequent images, taken with a high-speed camera (Sony PXW-FS5) just when the rod was entirely outside the channel) of 8.5 ± 0.5 m s^−1^ was estimated. The theoretical value of this upward velocity was calculated in the absence of dissipation to be equal to 12.1 m s^−1^. In addition to the vibration trapped inside the elastic rod after ejection (which is observed to have a small amplitude), there are two sources of dissipation explaining the difference between the data. The first, and less important, is due to the friction inside the channel which is not exactly null, but remains very small, because the rollers perform very well and the elastic rod was oiled.^5^ The second source is due to the fact that the passage of the rod induces a fast rotation in the rollers, so that in the channel prototype a non-negligible kinetic energy remains, stored as rotational inertia of the rollers.

**Figure 6.**
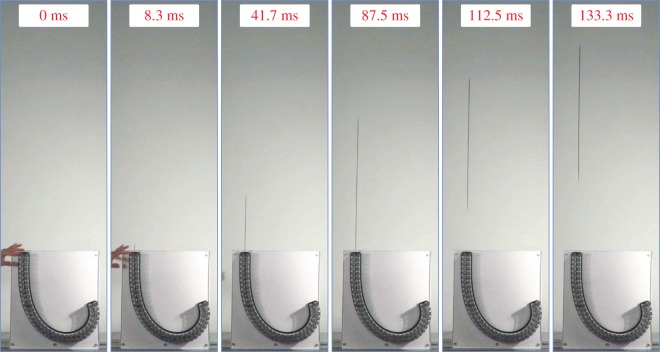
A sequence of frames (taken from a high-speed movie) showing the upward ejection of an elastic rod from the clothoid spiral channel shown in figure 5. The mean exit speed was found to be equal to 8.5 ± 0.5 m s^−1^ and was estimated considering two subsequent frames taken just when the rod was completely outside the channel. If the measured value of the velocity is compared with the theoretical one calculated in the absence of dissipation (12.1 m s^−1^), the efficiency of the device can be estimated. (Online version in colour.)

A sequence of frames (exported from a high-speed movie) is reported in figure 6 during a test (see the movie in the electronic supplementary material) showing how quickly the energy can be released as well as the efficiency of the propulsion.

### 4.2. Quantitative experiments

For a quantitative evaluation, the propulsive force *P* was measured by extracting the left edge (*s* = 0) of the rod from the channel at a constant low speed 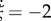 mm s^−1^, using a loading frame (Midi 10; Messphysik Materials Testing) placed horizontally and simultaneously acquiring the load (Mettler MT1041 load cell RC 200N connected to a NI CompactRio acquisition system).

With the purpose of *separately* verifying the different contributions to the propulsive force, three rods were tested that differ in the bending stiffness distribution *B*(*s*) provided by varying the width *b*(*s*), namely: a rod with constant stiffness (see figure 5*c*), a rod with a localized jump in the stiffness (see figure 5*d*) and a rod with a linearly varying stiffness (see figure 5*e*).

**Constant stiffness rod.** The effect of the channel curvature on the propulsive force has been measured for constant bending stiffness (*B*(*s*) = *B*_1_ = 1.211 × 10^6^ mm^4^, provided by *b*(*s*) = *b*_1_ = 21.1 mm) in the cases (B2) and (A) in figure 1. The propulsive force can be obtained from the particularization of equation (2.16) as4.2
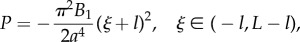
while from equation (2.20) the propulsive force is null for *ξ* ∈ (*L* − *l*, 0).

**Rod with a localized jump in stiffness.** The propulsive force has been measured when a rod with a finite jump in the bending stiffness (varying from *B*_1_ = 1.985 × 10^6^ mm^4^, provided by *b*_1_ = 34.5 mm, to *B*_2_ = 0.460 × 10^6^ mm^4^, provided by *b*_2_ = 8 mm, at the coordinate *l*_1_ = 1.1*L*) sweeps the channel (case B2 in figure 1),4.3
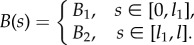
The propulsive force is theoretically predicted by equation (2.20) assuming *B*′(*s*) = 0 and taking 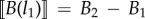 (where *β* = *B*_2_/*B*_1_ = 0.232) as4.4
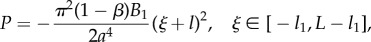
while from equation (2.20) the propulsive force is null for *ξ* ∈ (*L* − *l*, 0).

**Rod with a linearly varying stiffness.** The propulsive force generated during the movement of an elastic rod with a linearly varying bending stiffness (from *B*_1_ = 2.134 × 10^6^ mm^4^, provided by *b*_1_ = 34.5 mm, at the coordinate *s* = *l*_1_ = 1.1*L*, to *B*_2_ = 0.112 × 10^6^ mm^4^, provided by *b*_2_ = 2 mm, at the coordinate *s* = *l*_1_ + *d*, with *d* = 750 mm) has been measured (case B2 in figure 1),4.5
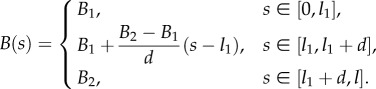
The propulsive force can be computed from equation (2.20) assuming *B*′(*s*) = (*β* − 1)*B*_1_/*d* (where *β* = *B*_2_/*B*_1_ = 0.054) and taking 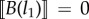 as4.6
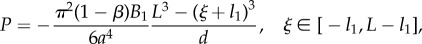
while from equation (2.20) the propulsive force is null for *ξ* ∈ (*L* − *l*, 0).

Although the channel is idealized as rigid and frictionless in the theoretical approach, a minimal amount of friction has to be expected in any practical realization. This friction has been measured by pulling through the channel a rod of constant bending stiffness, as shown in scheme B2 in figure 1, and finding that a non-null force was present (which was measured to oscillate between 3 and 4 N), instead of being null as theoretically predicted. This determination of the channel friction is important in our understanding of the experiments.

**Figure 7.**
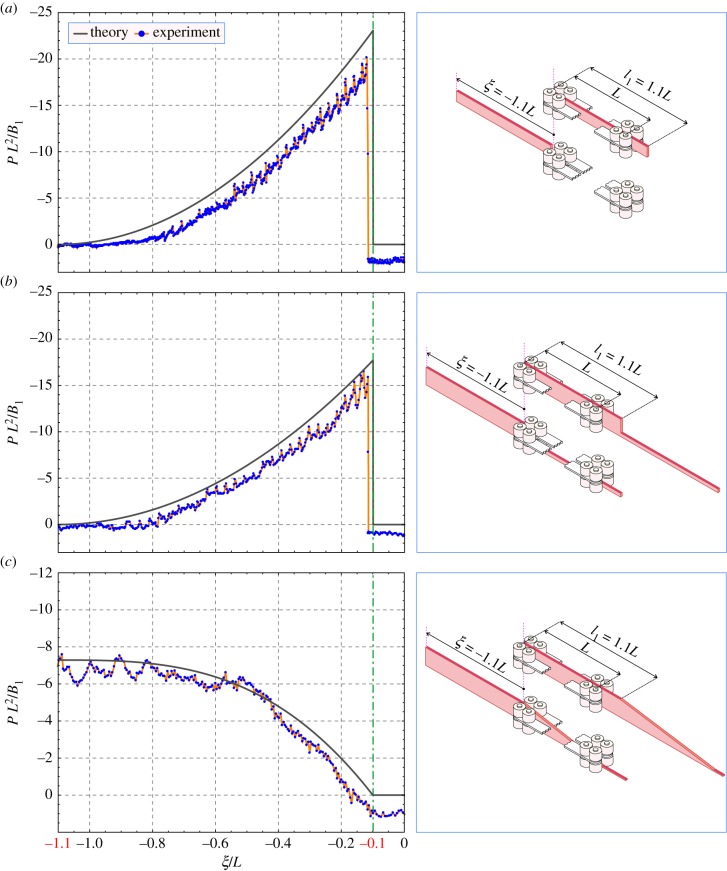
Experiments on elastic rods sliding within a frictionless channel. Three cases of constant (*a*), piecewise constant (*b*) and linearly varying (*c*) bending stiffness are considered. Left: experimental results (marked in blue) are close to the theoretical prediction (marked in black, and referring to equations (4.2), (4.4) and (4.6), respectively). The oscillation of the load is due to the gaps between the rollers; the fact that the experimental values are smaller than the predictions is due to the effect of friction, which is small, but not null. Right: sketch of the initial (*ξ*/*L* = 0) and final (*ξ*/*L* = − 1.1) configurations for the three cases of variation in the bending stiffness considered for the experiments (for simplicity, only a few rollers aligned on a straight line are reported). (Online version in colour.)

The results of the experiments describing the three cases of constant, discontinuous and linearly varying bending stiffness are, respectively, reported in figure 7*a*, 7*b* and 7*c*.

In figure 7, the propulsive force *P* (made dimensionless through multiplication by *L*^2^ and division by *B*_1_) is reported versus the configurational parameter *ξ* (made dimensionless through division by the channel length *L*). Note that the scale of the vertical axis in figure 7*c* is different from that in figure 7*a* and figure 7*b*.

In all the three cases, the graphs have to be read from right to left, because the tests were performed at decreasing *ξ*. The experiments have been designed to initiate from a situation of steady state (initiating from *ξ*/*L* = 0 and terminating at *ξ*/*L* = − 0.1), in which the propulsive force is theoretically null, *P* = 0.

In the two cases reported in figure 7*a*,*b*, the propulsive force jumps to a finite value when the rod's end (figure 7*a*) or the rod's discontinuity in *B* (figure 7*b*) enters the channel, *ξ*/*L* = − 0.1. Finally, at the exit from the channel, *ξ*/*L* = − 1.1, the propulsive force smoothly vanishes.

In the case reported in figure 7*c*, at decreasing *ξ*/*L*, the propulsive force *P* increases up to a maximum value attained at *ξ*/*L* = − 1.1 (and then would decay to zero, if the experiment were continued, which it was not). All theoretical predictions are reported with black curves and all experimental values with blue spots (joined with orange segments).

Although an immediate qualitative and quantitative agreement between theoretical predictions (black curves) and experimental values (marked in red) can be appreciated from figure 7, the discrepancies can be easily classified and explained as follows.

— The propulsive force displays an oscillation which is the consequence of the gaps between the rollers, an effect only partially alleviated by the Teflon layer. Note that the oscillations are more evident in the cases where either the rod's end (figure 7*a*), or the localized discontinuity in the rod's thickness (figure 7*b*) sweeps the channel and hits the rollers (see the detail in figure 5*a*). In the case when the rod has a continuous change in thickness *b* (figure 7*b*), the oscillations are strongly reduced (taking into account the different scale in the vertical axes of the graphs in the figure).
— The propulsive force is slightly reduced with respect to the expected theoretical value, even in the steady-state case, where it is negative rather than being null. This is the direct consequence of the small, but not null, friction in the channel that after normalization yields a spurious force *PL*^2^/*B*_1_ = 1.5 ± 0.5, almost exactly corresponding to the observed shift in the data.
— The propulsive force in figure 7*a*,*b* approaches zero faster than the theoretical prediction near *ξ*/*L* = − 1. This is simply due to the tolerance of 0.5 mm between the elastic rod and the rollers, so that the elastic rod reaches its undeformed configuration before it complete exits from the channel.

It can be concluded that the model of snake motion is definitively confirmed by experiments and that the propulsive force can be directly measured with a quasi-static electromechanical testing machine.

## 5. Conclusions, with a discussion on modelling snake motion

A model of serpentine motion, in which an elastic rod (the snake) moves inside a frictionless channel (the lateral restraints provided by projections from the ground or by transverse friction) through a release of (muscular) elastic energy, has been reviewed, corrected and extended, to cover situations occurring when the snake's body lies only partially inside the restraining channel and to explain tangential contact forces (so far ignored, but crucial to the propulsion). The presented theoretical results represent an extension and a clarification based on the concept of configurational force of the mathematical models introduced by Gray [1–3] and developed in several later contributions [5,7,13,15,30].

An experimental set-up has been designed, realized and tested, which allows for the first time direct measurement of the propulsive force in different situations, also involving a jump in the rod's bending stiffness (which a snake can obtain through a relaxation of muscles over a small zone of its body). Experimental results have been found to strongly confirm the theory, so that the developed experimental set-up is now available to directly measure the propulsive force on ‘prototypical snakes’ made up of elastic rods of different materials, stiffnesses and geometries. In this way difficulties related partially to mathematical modelling and partially to the use of living serpents, which are difficult to handle and to test, can be bypassed. The experiments can in fact be extended to situations involving friction (which is difficult to treat mathematically, because the transverse reactions in the channel are not known), or lubrication (the experimental equipment can be submerged in water or other liquids), or different stiffnesses (not only in the snake's body, but also in the channel transverse stiffness), and complex geometries of both the channel and the snake. Imagine, for instance, a snake moving against obstacles in an environment including partially submerged areas: using a physical model of such a situation, the developed experimental set-up would allow direct measurement of the propulsive force.

## Endnotes

1 In the absence of projections from the ground, for instance when the snake moves on a flat surface, friction transverse (longitudinal friction remains always detrimental to propulsion) to the body provides the necessary constraint. Therefore, friction at the contact between the snake's skin and the ground is strongly orthotropic, as shown in [5–7]. The model considered in the present article can be viewed as the extreme situation of orthotropy in which longitudinal and transverse friction are, respectively, null and infinite.

2 The elastic rod model was also used to explain concertina [18] and rectilinear movement, in the latter case introducing an axial deformation, so that propulsion is related to one-dimensional travelling waves [19] or diffusion [20–22]. In sharp contrast with the model for serpentine motion, the models for other movements are all based on friction between the animal and the ground, which has been analysed from several perspectives [23].

3 In the present analysis, the potential energy related to the weight of the bar is assumed constant during the motion. This condition is realized when the planar motion is orthogonal to the direction of gravity (as for a snake moving in a horizontal plane), but is also consistent when the variation in the gravitational energy is negligible with respect to the variations in elastic and kinetic energy occurring during the motion.

4 It is worth remarking that the assumption of a frictionless constraint does not imply that specific components of the channel reaction are zero, rather that the reaction components are restrained to a special setting providing null virtual work. Only in particular cases, such as those of rectilinear frictionless constraint or constrained rigid bodies, does the imposition of null virtual work imply the annihilation of some of the reaction components.

5 Note that during the ejection experiments the dynamic friction comes into play. The value of such a type of friction is much smaller than that reported later, as evaluated during quasi-static experiments.

## Data accessibility

Calculations were performed analytically. Experiments are reported in the figures and in the electronic supplementary material. Further data will be provided upon request.
